# An interspecies barrier to tetraploid complementation and chimera formation

**DOI:** 10.1038/s41598-018-33690-7

**Published:** 2018-10-16

**Authors:** Tomoyuki Yamaguchi, Hideyuki Sato, Toshihiro Kobayashi, Megumi Kato-itoh, Teppei Goto, Hiromasa Hara, Naoaki Mizuno, Ayaka Yanagida, Ayumi Umino, Sanae Hamanaka, Fabian Suchy, Hideki Masaki, Yasunori Ota, Masumi Hirabayashi, Hiromitsu Nakauchi

**Affiliations:** 1 Division of Stem Cell Therapy, Institute of Medical Science, University of Tokyo, Tokyo, 108-8639 Japan; 2 Center for Genetic Analysis of Behavior, National Institute for Physiological Sciences, Okazaki, Aichi 444-8787 Japan; 3 Department of Pathology, Research Hospital, Institute of Medical Science, University of Tokyo, Minato-ku, Tokyo 108-8639 Japan; 4 Institute for Stem Cell Biology and Regenerative Medicine, Stanford University School of Medicine, Stanford, CA 94305 USA; 5 Present Address: Center for Genetic Analysis of Behavior, National Institute for Physiological Sciences, Okazaki, Aichi 444-8787 Japan; 6 Present Address: Wellcome Trust-Medical Research Council Cambridge Stem Cell Institute, University of Cambridge, Cambridge, CB2 1QR UK

## Abstract

To study development of the conceptus in xenogeneic environments, we assessed interspecies chimera formation as well as tetraploid complementation between mouse and rat. Overall contribution of donor PSC-derived cells was lower in interspecies chimeras than in intraspecies chimeras, and high donor chimerism was associated with anomalies or embryonic death. Organ to organ variation in donor chimerism was greater in interspecies chimeras than in intraspecies chimeras, suggesting species-specific affinity differences among interacting molecules necessary for organogenesis. In interspecies tetraploid complementation, embryo development was near normal until the stage of placental formation, after which no embryos survived.

## Introduction

Advances in chimera and stem cell technology has provided intriguing tools with many basic and translational applications. Since chimeras have at least two cell populations with different genetic backgrounds, they can be used to compare cell potency and function, or probe signaling pathways during development. Generation of chimeras using PSCs is well-established for intraspecies applications^1–7^, however is more difficult in an interspecies setting. One exciting interspecies application exploits mouse-rat chimerism to generate PSC-derived organs by blastocyst complementation^8^. Injection of rat induced PSCs (iPSCs) into Pdx1^−/−^ mouse blastocysts successfully generated a rat pancreas in a mouse, demonstrating that PSCs can contribute to xenogenic embryo development with postnatal survival. These interspecies chimeras, however, exhibited donor chimerism lower than that of intraspecies chimeras. Interspecies chimeras with high chimerism suffered embryonic lethality and were malformed. These findings indicate the presence of a barrier to interspecies chimera formation.

In mammals, fetal development requires support from the placenta, which regulates transport of gases, nutrients, antibodies and placenta derived factors to/from the fetus. This also entails regulating continuous, appropriate cell growth and differentiation consequent upon complex cell-to-cell interactions. We suspected that these interactions are flawed or distorted in interspecies chimeras, and that these discrepancies constituted the barrier to interspecies chimera generation that our work had encountered. To better understand this barrier, we used interspecies tetraploid complementation to investigate the effect of xenogenic extraembryonic tissue on development of PSC-derived embryos.

Tetraploid complementation is a technique used to generate fetuses entirely derived of pluripotent stem cells (PSCs). Although PSCs such as embryonic stem cells (ESCs) or induced PSCs (iPSCs) can differentiate into any cell of the embryo proper, they lost their ability to differentiate into most placental tissues, thus cannot alone assemble into an embryo capable of implantation. However, fetuses entirely derived from PSCs can be generated if PSCs are injected into early tetraploid (4N) embryos produced by electrofusion of a 2-cell zygote. Unlike the placenta, the embryo proper cannot develop normally with 4N cells. Tetraploid complementation is therefore a technique that restricts the 4N host embryo to extraembryonic lineages, creating an “embryo proper niche” that can be filled with donor PSCs^9^. The offspring generated by tetraploid complementation are distinct from chimeras—although the conceptus is chimeric, the embryo proper is derived from a single genetic background.

Limitations of interspecies chimeras, including cell contribution and tissue distribution, have not been thoroughly explored. Identifying these limits will not only highlight the temporal and spatial occurrence of a xenogenic barrier, but may also provide clues regarding which cell types and organs are most amenable to interspecies chimerism and organogenesis. Here, we first use tetraploid complementation to explore development of PSC derived embryos with xenogenic extraembryonic tissues. We then probe the functional limits of various PSCs regarding their ability to contribute to intra- and interspecies chimeras across multiple tissues and developmental timepoints.

## Results

### Development of PSC-derived embryos in a xenogenic environment

To examine the developmental potential of PSC-derived embryos in a xenogenic environment, we first injected rat PSCs (rPSCs) into mouse tetraploid embryos.

**Figure 1:**
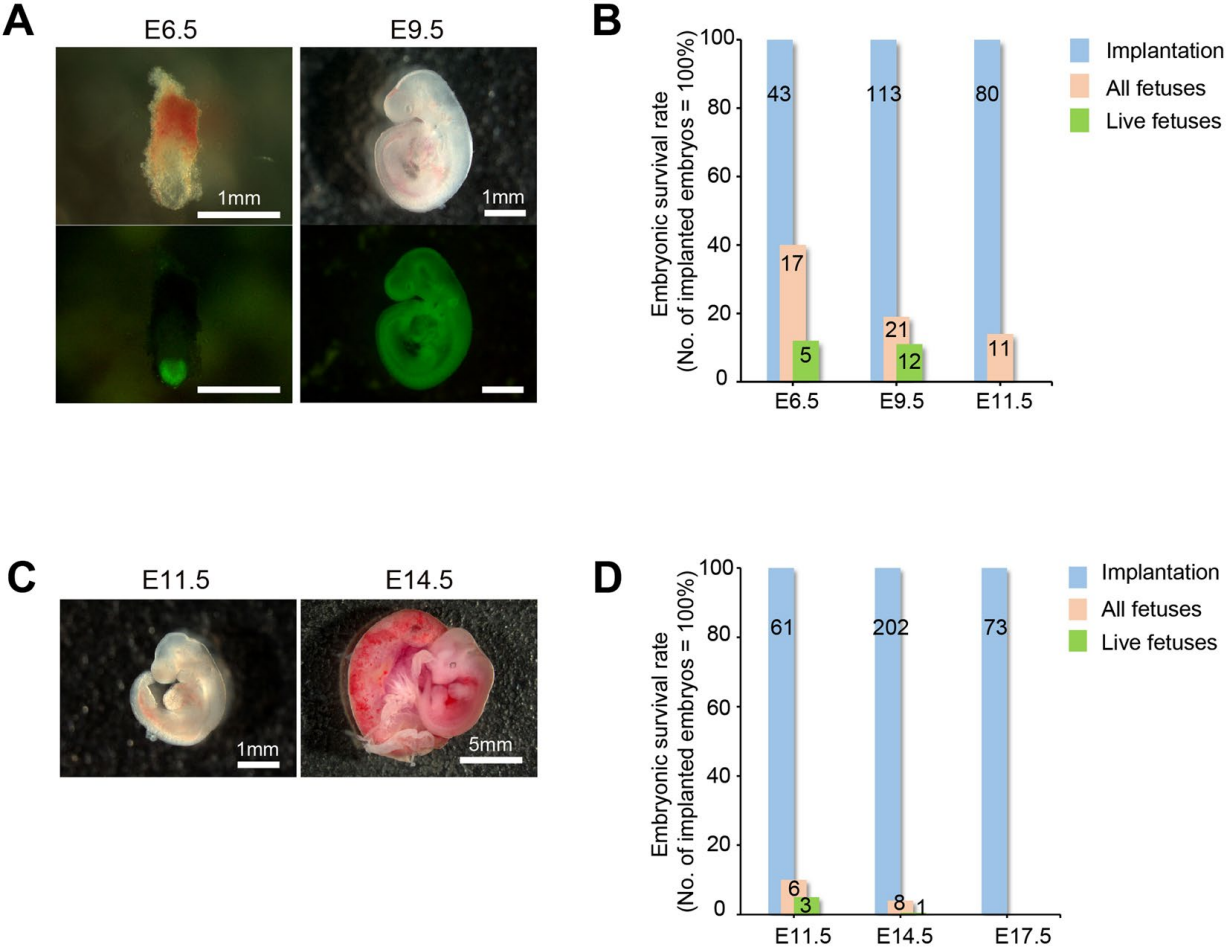
Generation of rat or mouse embryo by interspecies tetraploid complementation (**A**,**C**) Morphology of embryos generated by interspecies tetraploid complementation. Bright field images (upper) and fluorescent images (lower) of E6.5 (left) and E9.5 (right) rat PSC-derived embryos (**A**) (scale bars: 1 mm). Bright field images of E11.5 (left) and E14.5 (right) mouse PSC-derived embryos (**C**) (scale bars: 1 mm, left, and 5 mm, right). (**B**,**D**) Survival rates at E6.5, E9.5, and E11.5 of rat embryos (**B**) and at E11.5, E14.5, and E17.5 of mouse embryos (**D**) generated by interspecies tetraploid complementation. The rate of implantation is taken as 100%. Blue bar, pink bar, and green bar represent percentages of implantation (blue), of all embryos including degenerating embryos (pink), and of live embryos (green). The numbers in the bar are the actual numbers of implanted embryos (blue), of all embryos (pink), and of live embryos (green).

After embryo transfer, we assessed development of the PSC-derived embryos at several stages. Injection of enhanced green fluorescent protein (EGFP) expressing rat iPSCs (T1-3) yielded, at embryonic day [E] 6.5, epiblasts that were composed entirely of EGFP^+^ rat iPSC-derived cells and were surrounded by EGFP^−^ mouse extraembryonic tissues (Fig. 1A). At E9.5, embryos enclosed in mouse extraembryonic tissues expressed EGFP ubiquitously from head to tail (Fig. 1A). 12 live embryos were curved ventrally, with red blood cells in the aortic region (Fig. 1A and Table 1) and those embryos were correspond to E10.5 to 11 stage of rat embryo based on their forelimb bud morphology. Immunohistological study and flow cytometric analysis clearly showed that these embryos were entirely composed of EGFP^+^ rat iPSC-derived cells (Fig. S1A,B). Immunofluorescent staining of embryonic tissue with antibodies against markers for the three representative germ layers revealed that rat iPSC-derived embryos had beta III-tubulin^+^ neural cells, forkhead box protein A2 (FoxA2)^+^ gut-endoderm cells, and platelet endothelial cell adhesion molecule 1 (PECAM-1)^+^ mesoderm-oriented blood vessels (Fig. S1C). These data strongly suggest that rat iPSC-derived cells had normally differentiated into various types of tissues even in a mouse environment until E9.5.

**Table 1:** Results of interspecies tetraploid complementation by injection of rat PSCs into mouse tetraploid embryos.

Donor cell name	Donor strain	Donor cell type	Tetraploid embryo strain	Stage of analysis	No. of implanted embryos	No. of embryos (%)^a^	No. of PSC-derived live embryos or pups (%)^b^
T1-3	Wistar	iPSC	BDF1 × C57BL/6	E6.5	19	10 (53)	4 (21)
				E9.5	65	18 (28)	9 (14)
				E11.5	59	7 (12)	0 (0)
				E12.5	89	0 (0)	0 (0)
				E13.5	72	0 (0)	0 (0)
WI3i-1	Wistar	ESC	BDF1 × C57BL/6	E6.5	24	7 (29)	1 (4)
				E9.5	11	0 (0)	0 (0)
				E9.5	6	0 (0)	0 (0)
BLK-RT2	DA × Wistar	ESC	BDF1 × C57BL/6	E9.5	31	3 (10)	3 (10)
				E11.5	21	4 (19)	0 (0)

^a^Frequencies of embryos (%) were determined by dividing the number of implanted embryos by the number of embryos.

^b^Frequencies of PSC-derived live embryos or pups (%) were determined by dividing the number of implanted embryos by the numbers of PSC-derived live embryos or pups.

To check for further developmental potential of rat PSCs in mice, we analyzed embryos at E11.5–13.5. However, no live embryos were found at E11.5 (Fig. 1B and Table 1). At this timepoint the embryos exhibited substantial tissue degeneration (Fig. S1D) when compared with E11.5 embryos generated by injection of rat PSCs into rat tetraploid embryos^10^. On the other hand, the mouse tetraploid embryos could support full term development with mouse PSCs, confirming their developmental potential in an intraspecies setting (Fig. S2). This indicated that the mouse placental/maternal environment was not competent to support development of rat PSCs beyond E9.5.

We next attempted to develop mouse embryos in a rat environment by injection of tetraploid-competent mouse PSCs into rat tetraploid embryos. Although we could not obtain live pups with interspecies tetraploid complementation compared with intraspecies (Table 2 and Fig. S2B), mouse PSC-derived embryos were able to develop until E11.5, were curved ventrally with red blood cells in the aortic region and those embryos were correspond to E9.5 stage of mouse embryo based on their forelimb bud morphology (Fig. 1C). Moreover, when we analyzed embryos at E14.5, we found further developed mouse ESC-derived embryos in rat (Fig. 1D).

**Table 2:** Results of interspecies tetraploid complementation by injection of mouse PSCs into rat tetraploid embryos.

Donor cell name	Donor strain	Donor cell type	Tetraploid embryo strain	Stage of analysis	No. of implanted embryos (%)	No. of embryos (%)^a^	No. of PSC-derived live embryos or pups (%)^b^
GT3.2	C57BL/6	iPSC	Wistar	E11.5	33	1 (3)	1 (3)
K3	129sv × C57BL/6	ESC	Wistar	E11.5	28	5 (18)	2 (7)
				E14.5	101	5 (5)	1 (1)
				E17.5	73	0 (0)	0 (0)
K3	129sv × C57BL/6	ESC	Wistar × CAG-Venus^TG/TG^	E13.5	79	3 (4)	2 (3)
				E14.5	101	3 (3)	0 (0)

^a^Frequencies of embryos (%) were determined by dividing the number of implanted embryos by the number of embryos.

^b^Frequencies of PSC-derived live embryos or pups (%) were determined by dividing the number of implanted embryos by the numbers of PSC-derived live embryos or pups.

These embryos were mostly morphologically normal and showed pulsation of the heart (data not shown). These data clearly indicate that rat tetraploid embryos can support the development of mouse PSC-derived embryos until the E14.5 stage.

We followed the development of mouse embryos in the rat environment for three more days, however, we could not detect any live mouse embryos at E17.5 (Fig. 1D and Table 2). This limitation resembles that encountered with rat PSC-derived embryos in the mouse environment.

### Ability of PSCs to contribute to interspecies chimeras

**Figure 2:**
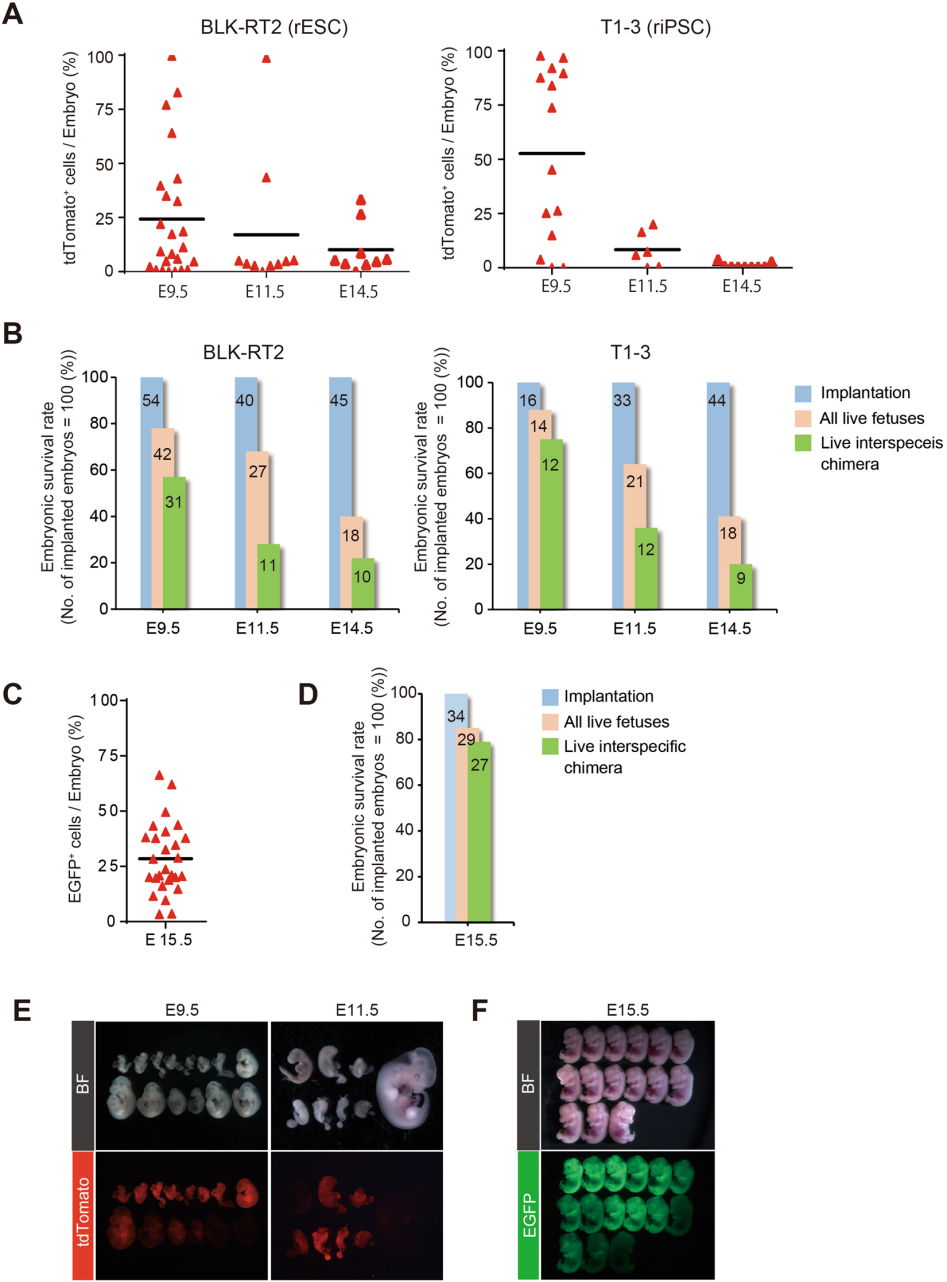
Contribution of rat or mouse PSCs to interspecies chimeras. (**A**,**C**) Chimerism of rat ESC (left) and rat iPSC (right) derivatives at E9.5, E11.5, and E14.5 in interspecies chimeras (**A**) and mouse ESC derivatives at E15.5 in interspecies chimeras (**C**). (**B**) Survival rates at E9.5, E11.5, and E14.5 of interspecies chimeras generated by injection of rat ESCs (left) and rat iPSCs (right) into mouse blastocysts (**B**) survival rate at E15.5 of interspecies chimeras generated by injection of mouse ESCs into rat blastocysts. (**D**) Blue bar, pink bar, and green bar represent percentages of implantation (blue), of all embryos including degenerating embryos (pink), and of live embryos (green). The numbers in the bar are the actual numbers of implanted embryos (blue), of all embryos (pink), and of live embryos (green). (**E**) Bright field (upper) and fluorescence-microscopy images (lower) of E9.5 (left) and E11.5 (right) interspecies chimeras generated by injecting rat PSCs (**E**) and E15.5 interspecies chimeras generated by injecting mouse ESCs (**F**).

To investigate the limits of mouse and rat PSC contributions to forming interspecies chimeras, we generated rat-mouse interspecies chimeras by injecting rat or mouse PSCs into mouse or rat blastocysts and analyzed whole-embryo chimerism at several different developmental stages (Table 3). The average chimerism of E9.5 embryos generated by injection of rat PSCs into mouse blastocysts was 24.3% using ESCs and 52.7% using iPSCs. In both groups, chimerism declined to less than 11% as the developmental stage advanced (Fig. 2A**)** as did the chimera survival rate. At E9.5, survival was 57% for the ESC group and 75% for the iPSC group, falling to less than 25% at E14.5 in both group (Fig. 2B).

**Table 3:** Results of intraspecies chimera formation by injection of rat or mouse PSCs into mouse or rat blastocysts.

Donor cell name	Donor strain	Donor cell type	Blastocysts strain	Stage of analysis	No. of implanted embryos (%)	No. of embryos (%)^a^	No. of PSC-derived live embryos or pups (%)^b^
BLK-RT2	Wistar	Rat ESC	BDF1 × C57BL/6 (Mouse)	E9.5	54	42 (78)	31 (57)
				E11.5	40	27 (68)	11 (28)
				E14.5	45	18 (40)	10 (22)
T1-3	Wistar	Rat iPSC	BDF1 × C57BL/6 (Mouse)	E9.5	16	14 (88)	12 (75)
				E11.5	33	21 (64)	12 (36)
				E14.5	44	18 (41)	9 (20)
SGE2	C57BL/6	Mouse ESC	Wistar (Rat)	E15.5	34	29 (85)	27 (79)

^**a**^Frequencies of embryos (%) were determined by dividing the number of implanted embryos by the number of embryos.

^b^Frequencies of PSC-derived live embryos or pups (%) were determined by dividing the number of implanted embryos by the numbers of PSC-derived live embryos or pups.

Morphological analysis revealed normal development at E9.5 in 50% of chimeric embryos generated by injection of rat PSCs into mouse blastocysts. However, development was abnormal in almost all embryos (88%) at E11.5, with highly chimeric embryos tending to be malformed (Fig. 2E). By contrast, average chimerism of embryos and survival rate of chimeras generated by injection of mouse PSCs into rat blastocysts were high (28% and 79% respectively) at E15.5 (Fig. 2C,D) and the chimeric embryos were morphologically normal (Fig. 2F). These results indicate that mouse and rat PSCs have similar abilities to contribute to chimera formation when assessed in early embryonic interspecies chimeras, and that mouse PSCs maintain this contribution level during the prenatal period. However, interspecies chimeras with a high contribution of rat PSCs were absorbed as development advanced.

### Developmental capacity of PSC derived cells in xenogenic organs

**Figure 3:**
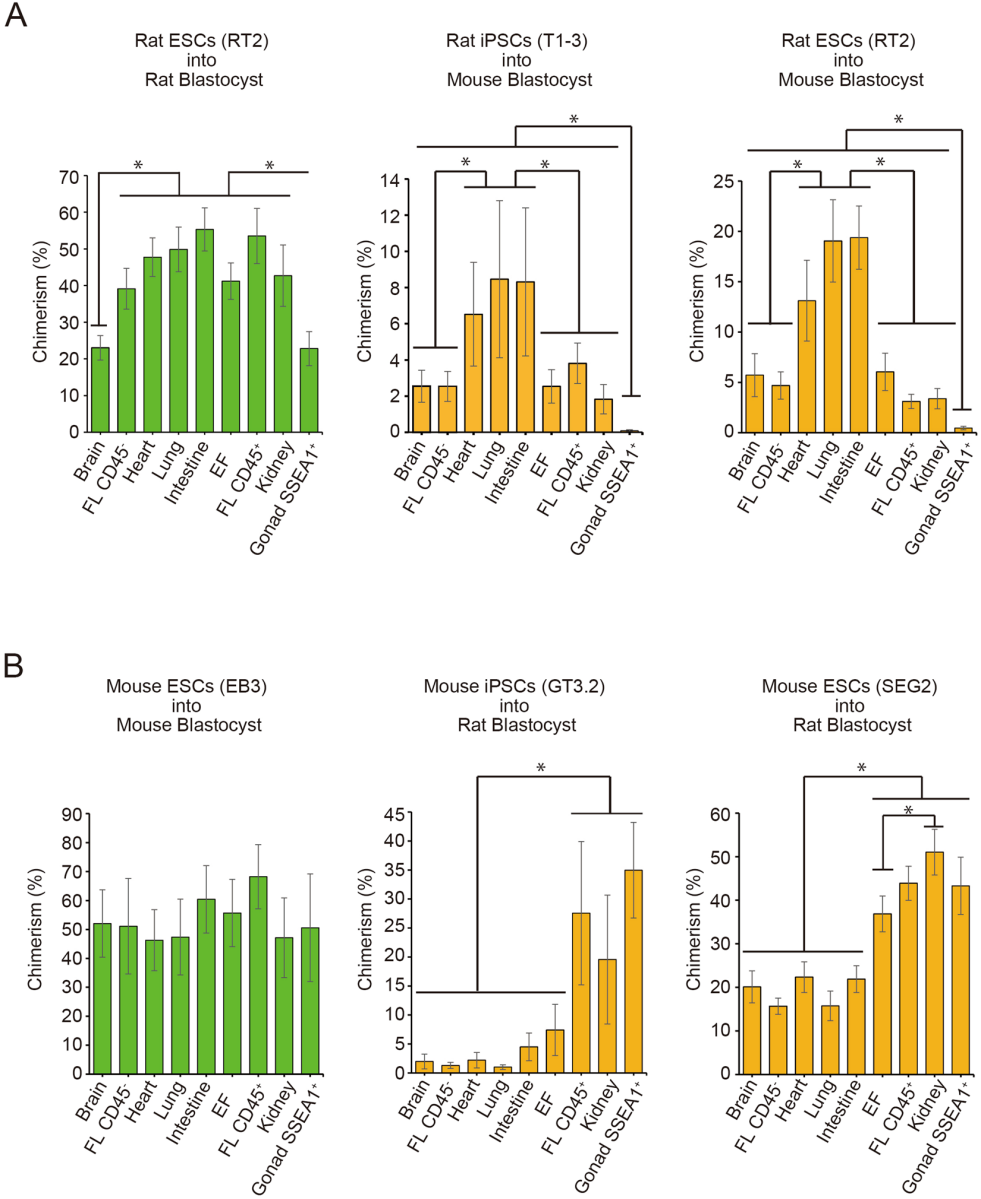
Contributions of rat or mouse PSCs to the organs of interspecies chimeras Donor PSC derivative chimerism in brain, CD45^−^ hepatic cells in fetal liver (FL CD45^−^), heart, lung, intestine, embryonic fibroblast (EF), CD45^+^ hematopoietic cells in fetal liver (FL CD45^+^), kidney, and SSEA1^+^ gonad cells (Gonad SSEA1^+^) of E14 to E15 chimeras. (**A**) Chimerism of rat ESC derivatives in the organs of intraspecies chimeras (left; n = 6), chimerism of rat iPSC derivatives in the organs of interspecies chimeras (middle; n = 10), and chimerism of rat ESC derivatives in the organs of interspecies chimeras (right; n = 10). (Mean values ± SEM were obtained from 6 or 10 independent experiments. ^*^P < 0.05; Student’s *t*-test.) (**B**) Chimerism of mouse ESC derivatives in the organs of intraspecies chimeras (left; n = 5), chimerism of mouse iPSCs derivatives in the organs of interspecies chimeras (middle; n = 5), and chimerism of mouse ESCs derivatives in the organs of interspecies chimeras (right; n = 23 to 28). (Mean values ± SEM were obtained from 5, 23, or 28 independent experiments. ^*^P < 0.05; Student’s *t*-test.)

We also analyzed chimerism in various organs at E14 to E15. In embryos generated by injection of rat ESCs into mouse blastocysts, relatively high chimerism was observed in heart (13.1 ± 4.0%), lung (19.0 ± 4.1%), and intestine (19.4 ± 3.1%). The same trend was observed in rat iPSC-injected chimeric embryos (heart 6.5 ± 2.9%, lung 8.5 ± 4.3%), and intestine 8.3 ± 0.9%) (Figs 3A and S3A). To determine if the rat PSC distribution differences among organs was caused by aberrant methylation patterns in the rat PSCs^10^, we injected rat ICMs into mouse blastocysts and analyzed chimerism in various organs of the embryos produced. Although overall chimerism was higher than in embryos generated by injecting rat PSCs, contribution patterns were similar except for brain (Fig. S4).

In embryos generated by injection of mouse ESCs into rat blastocysts, relatively high chimerism was observed in embryonic fibroblasts (EF; 36.9 ± 4.1%), kidney (51.0 ± 5.2%), CD45^+^ hematopoietic cells in fetal liver (FL CD45^+^; 43.9 ± 3.9%) and stage-specific embryonic antigen 1 (SSEA1)^+^ gonadal cells (Gonad SSEA1^+^; 43.3 ± 6.6%). Conversely, relatively low chimerism was observed in brain (20.1 ± 3.7%), CD45^−^ hepatic cells in fetal liver (FL CD45^−^; 15.7 ± 1.9%), heart (22.3 ± 3.5%), lung (15.8 ± 3.4%), and intestine (21.9 ± 3.1%) (Figs 3B and S3B). The same trend was observed in embryos generated by injection of mouse iPSCs into rat blastocysts. Relatively high chimerism was observed in EF (7.4 ± 4.4%), kidney (19.6 ± 11.1%), FL CD45^+^ (27.6 ± 12.4%), and Gonad SSEA1^+^ (35.0 ± 8.2%) and low chimerism was observed in brain (2.0 ± 1.3%), FL CD45^−^ (1.3 ± 0.5%), heart (2.2 ± 1.3%), lung (1.0 ± 0.4%), and intestine (4.5 ± 2.4%) (Figs 3B and S3B).

These data indicate that developmental limitations of PSCs in interspecies chimeras differ in each organ, and that the contribution pattern varies with species of injected PSC and the corresponding interspecies blastocyst. Moreover, trends in contribution rates of rat PSCs to each organ except the brain were not due to aberrant methylation patterns.

### Tissue and organ abnormalities in interspecies chimeras

**Figure 4:**
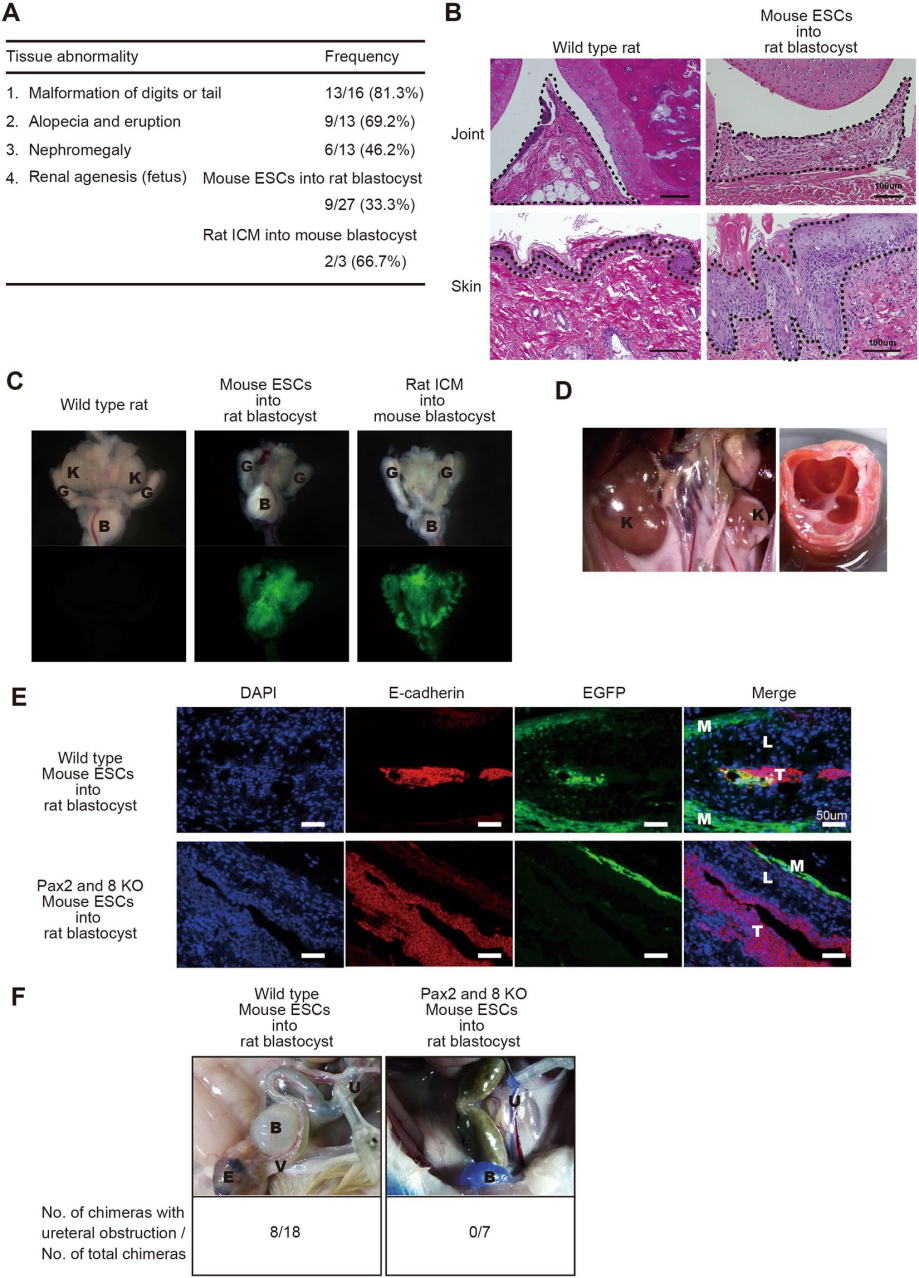
Abnormalities in interspecies chimeras (**A**) Frequency of macroscopic abnormalities observed in adult or fetal interspecies chimeras. (**B**) Photomicrographs, sections of digit’s joint of wild type rat (upper left) and interspecies chimera generated by injection of mouse ESCs into rat blastocyst (upper right). The area inside the dot line represents synovial. Sections of skin of wild type rat (lower left) and interspecies chimera generated by injection of mouse ESCs into rat blastocyst (lower right). The area inside the dot line represents epidermis. Hematoxylin and eosin (HE); scale bars: 100 um. (**C**) Bright field images (upper) and fluorescent images (lower) of urinary organs in wild type rat (left), interspecies chimera generated by injection of mouse ESCs into rat blastocyst (middle), and interspecies chimera generated by injection of rat inner cell mass (ICM) into mouse blastocyst (right). Kidney (K), genital ridge (G) and bladder (**B**) are abbreviated as indicated. Note absence of kidney, middle and right panels. (**D**) Hypertrophic kidney in interspecies chimera generated by injection of mouse ESCs into rat blastocyst (left). Dissected hypertrophic kidney (right). Kidney (K) is abbreviated as indicated. (**E**) Immunofluorescence appearance of nephric duct of interspecies chimera generated by injection of wild type mouse ESCs into rat blastocyst (upper) and of interspecies chimera generated by injection of Pax2 and 8 mutated mouse ESCs into rat blastocyst (lower). Sections were stained with antibodies against e-cadherin (red) and EGFP (green). Cell nuclei were stained with DAPI. Muscle layer (M), lamina propria (L) and transitional epithelium (T) are abbreviated as indicated. Scale bars: 50 um (**F**) Urine flow testing of interspecies chimera generated by injection of wild type mouse ESCs into rat blastocyst (left) and interspecies chimera generated by injection of Pax2 and 8 mutated mouse ESCs (right). Lower column represents the results of flow testing (number of chimera with ureteral obstruction/number of total chimera).

While generating interspecies chimeras, we observed anomalies in several tissues and organs, especially in adult interspecies chimeras generated by injection of mPSCs. Most frequent was malformation of digits or tail (81.3% of adult interspecies chimeras) (Fig. 4A,B). Alopecia and eruption (69.2%) as well as nephromegaly (46.2%), were also common (Fig. 4A,B,D). Renal agenesis occurred in fetal interspecies chimeras generated by both mPSC injection (33.3%) and rat ICM injection (66.7%) (Fig. 4A,C).

On histopathologic examination, lymphoid cell infiltration and dilated capillaries were present in malformed digits and tails. As no bone abnormality was observed, the malformations were ascribed to synovitis (Fig. 4B). In skin, dyskeratosis, blister formation, acanthosis, lymphoid infiltration and hypertrophy of the epidermis indicated dermatitis (Fig. 4B). Urine flow testing, with microscopy of the ureter and the bladder, revealed that nephromegaly was caused by hydronephrosis induced by ureteral obstruction (Fig. 4F). To examine whether ureteral obstruction was caused by existence of xenogeneic cells in nephric duct during the development, we generated interspecies chimera by injection of Pax2 and Pax8 double knockout ES cells into Rat blastocyst. Previous reports showed that Pax2 and Pax8 double deficient mouse embryo were unable to form nephric duct because of deficient of the mesenchymal-epithelial transitions of the intermediate mesoderm^11^. Immunofluorescent staining of nephric duct with antibodies against markers for transitional epithelium showed that Pax2 and Pax8 double mutant cells did not contribute transitional epithelium in interspecies chimera. Urine flow testing revealed that no ureteral obstruction was observed in Pax2 and Pax8 double mutant ESCs injected interspecies chimera (Fig. 4F). These results indicate that developmental defects in interspecies chimera was caused by existence of xenogenic cells during the development.

## Discussion

In this study, we demonstrated the existence of a barrier to interspecies tetraploid complementation and to interspecies chimera formation.

In mouse and rat development, placenta formation begins around E9.5 and ends by E14. During this time, the placenta acquires the important role of sustaining late-stage embryo development. In both the mouse and rat interspecies tetraploid complementation experiments, the embryo proper could develop until at least E9.5. It was not until after initiation of placentation that the xenogenic barrier became explicit, ultimately resulting in embryonic death.

In our interspecies tetraploid complementation setting, the rat placenta formed nicely by E14.5 as shown in Fig. 1C. The size and blood flow of the placenta appeared normal, however it did not support development of late-stage mouse embryos. Whole embryo culture *in vitro* is similarly limited, sustaining development without the placenta to around E11–E13.5^12^. Therefore, mouse embryonic death in the case of interspecies tetraploid complementation is likely due to inappropriate function of the placenta. Functional aspects such as exchange of gases and supply of nutrition or other supportive factors are likely insufficient, particularly if those factors are species-specific.

By contrast, when rat iPSCs were injected into mouse tetraploid blastocysts, none of the rat embryos survived to E11.5. Placental function therefore cannot be assessed at this timepoint, as the placenta is not fully developed. Regardless, early incompatibilities among the developing xenogenic placenta and embryo proper could be the cause of embryonic lethality since development is normal until the onset of placentation.

Similar developmental arrest and abnormalities were found in interspecies chimeras generated by injecting rat PSCs into mouse blastocysts. As shown in Supplementary Fig. 4, chimerism was higher when rat ICM cells instead of rat PSCs were injected into mouse blastocysts. Although these rat PSCs generated higher chimerism when injected into rat blastocysts, these data suggest that they lost or failed to utilize some of the properties of ICM cells necessary in the xenogenic environment. One possible reason is an aberrant epigenetic status of the injected rat PSCs. Methylation patterns of differentially methylated regions in a normally developing rat differ not only from rat PSC-derived embryos, but also from the PSCs themselves^10^. Aberrant methylation status in mouse ESCs also is associated with abnormalities and lethality in chimeric mice^13^. This phenomenon in rat PSCs might also be developmentally lethal in the xenogenic environment.

Interestingly, we found drastic organ-to-organ variation in donor chimerism. Environmental factors thus must influence differentiation of xenogenic PSC-derived cells. These data suggest species-specific affinity differences among interacting molecules necessary for development of germ cells.

Various malformations and anomalies were observed in interspecies chimeras. The frequencies of malformation and anomalies appeared proportional to the level of donor chimerism. Because these malformations were observed even when ICM cells were injected, and because malformations were rare in intraspecies chimeras and no nephric duct abnormality was observed when the nephric duct was not interspecies-chimeric, this was likely not due to abnormalities in PSCs but instead to xenogenic incompatibilities such as inappropriate timing and site for cellular interactions, mismatched ligand-receptor interactions, affinity differences in adhesion molecules, and other cell intrinsic or extrinsic factors.

The malformations and anomalies observed in these chimeras may therefore provide important clues to understanding organogenesis at the molecular level. Comprehensive understanding of these molecular pathways could not only unlock mysteries to evolutionary and developmental biology, but also provide tools for regenerative medicine and ultimately human organogenesis.

## Materials and Methods

### Animals

All mice and rats were maintained under specific pathogen-free conditions. All animal experiments were performed in accordance with the guidelines of the University of Tokyo and the National Institute for Physiological Sciences, Japan.

All animal and recombinant DNA experimental protocols were approved by The University of Tokyo Animal Care and Use Committee (no. A16-22, A16-61 and A16-66).

### Cell culture and isolation of ICM

Maintenance of mouse and rat pluripotent stem cells is described^8,10,14–17^.

For preparation of donor ICM, immunosurgery of rat blastocysts was performed as described^18^. Briefly, the zonae pellucidae were removed from E5.5 rat blastocysts with acidic Tyrode’s solution (Sigma-Aldrich, St. Louis, MO, USA). 20% anti-rat whole serum (ROCKLAND Immunochemicals INC. Limerick, PA, USA; 112-4101)/DMEM (Sigma-Aldrich) for 3 hr. The blastocysts were washed once with DMEM and incubated for 20 min in 100% rat serum as the complement source. The trophectoderm cells were removed by gentle pipetting.

### Tetraploid complementation

Tetraploid complementation was performed as described^10,15^. Briefly, for production of tetraploid embryos, 2-cell stage diploid embryos were collected in M2 medium (Merck Millipore, Darmstadt, Germany, MR-015P-D) from oviducts of mice 1.5 days postcoitum (dpc). These embryos were washed 3 times with medium containing 0.01% polyvinyl alcohol (Sigma-Aldrich; P8136), 280 mM mannitol (Sigma-Aldrich; M4125), 0.5 mM Hepes (Sigma-Aldrich; H4034), and 0.15 mM MgSO_4_ (Wako, Osaka, Japan; 131-00405). Electrofusion of blastomeres to produce tetraploid embryos was carried out using a DC pulse (100 V/mm, 30 µsec, 1 time) followed by application of AC pulses (5 V/mm, 10 sec) using an ECM 2001 (BTX, Holliston, MA). These tetraploid embryos were transferred into potassium simplex optimized medium with amino acids (KSOM-AA) (Merck Millipore; MR-020P-D) and were cultured for 24–48 h for 4-cell/morula or blastocyst injection.

For micro-manipulation, ESCs/iPSCs were trypsinized and suspended in their culture medium. A piezo-driven micro-manipulator (Prime Tech, Tokyo, Japan) was used to drill the zona pellucida under the microscope and 5–10 cells were introduced into the perivitelline space of 4-cell/morula stage tetraploid embryos. ICMs were introduced into blastocyst cavities near the inner cell mass. After injection, tetraploid embryos underwent follow-up culture to blastocyst stage. The blastocysts then were transferred into the uteri of pseudopregnant recipient ICR female mice (2.5 dpc).

### Intraspecies and interspecies chimeras

Generation of intraspecies and interspecies chimera was performed as described^8,16^. Briefly, mouse 8-cell/morula stage embryos were collected in M2 medium from oviduct and uterus of mice 2.5 dpc. These embryos were transferred into KSOM-AA and were cultured for 24 hr for blastocyst injection.

Wild-type rat blastocysts were collected in a bicarbonate-buffered medium composed of Roswell Park Memorial Institute medium (RPMI) 1640, Eagle’s solution, and Ham’s F12 containing 18% FBS medium^19^ from oviduct and uterus of rats 4.5 dpc. These embryos were transferred into modified rat 1-cell embryo culture medium^20^ containing 80 mM NaCl (Wako) and 0.1% polyvinyl alcohol (Sigma) and were cultured for about 1 hr until injection.

For micromanipulation, ESCs or iPSCs were trypsinized and suspended in ESC or iPSC culture medium. A piezo-driven micromanipulator (Prime Tech, Tokyo, Japan) was used to drill the zona pellucida and trophectoderm under the microscope and 5–10 ESCs or iPSCs were introduced into blastocyst cavities near the inner cell mass. After blastocyst injection, embryos underwent follow-up culture for 1–2 hr. Mouse blastocysts then were transferred into the uteri of pseudopregnant recipient ICR female mice (2.5 dpc) and rat blastocysts were transferred into the uteri of pseudopregnant recipient Wistar female rats (3.5 dpc). To generate interspecies chimeras for morphologic analysis, we used blastocysts obtained from wild type mice, wild type rats, and *Pdx1* heterozygous mutant rats^21^.

### Chimerism analysis

Whole embryos or organs (brain, mouse embryonic fibroblast (MEF), heart, lung, intestine, kidney, fetal liver cells, or gonadal cells) isolated from E10.5–11.5 or E14.5–15.5 embryos were dissociated with collagenase type IA (Sigma). Dissociated cells were analyzed for fluorescent protein expression by flow cytometry using a FACS Cant II (BD Biosciences, San Jose, CA, USA).

### Immunohistochemistry

Immunohistochemistry was performed as described^8,15^. Briefly, fetuses were fixed with 4% paraformaldehyde and embedded in paraffin or, for frozen sections, in Optimal Cutting Temperature (O.C.T.) compound (Sakura Finetek, Tokyo, Japan). Paraffin-embedded sections were deparaffinized with xylene and hydrated with graded ethanols. An autoclave was used for antigen retrieval. Paraffin and frozen sections were stained immunohistochemically. Briefly, each section was incubated with primary antibody overnight at 4 °C and with secondary antibody for 1 hr at RT. Primary antibodies against EGFP (rabbit IgG, Invitrogen. clone No, A11122.; rat IgG, NACALAI TESQUE, Kyoto, Japan. clone No, GF090R.; goat IgG, abcam, Cambridge, UK. Cat No, ab6673), Foxa2 (goat IgG, Santa Cruz Biotechnology, Dallas, Tx, USA. Clone No, SC6554), Tuj1, PECAM1 (rat IgG, BD, San Diego, CA, USA. Clone No, TLD-3A12) and E-cadherin (Mouse IgG, BD Biosciences, San Jose, CA, USA Clone No, 36/E-cadherin) were used. Secondary antibodies used were Alexa488-, Alexa 568-, and Alexa 647-conjugated and were directed against rabbit, rat, and goat IgG (Invitrogen). After antibody treatment, samples were stained with 4′,6-diamidino-2-phenylindole (DAPI) to mark nuclei and were observed using an all-in-one BZ-9000 fluorescence microscope (Keyence, Osaka, Japan). Adult tissues were fixed with formalin and embedded in paraffin. Paraffin sections were stained with hematoxylin and eosin (HE) for light microscopy.

### Genome editing

Pax2 and 8 genes in mouse ESCs were mutated with CRISPR/Cas9 system by biallelic deletion of 588 bp in exon2 (Pax2) and 522 bp in exon2 (Pax8)^22,23^. The sequence of gRNAs were GTGTCAGCAAAATCCTGGGC (gRNA1) and GCCAAACCTGCGGCGCAGGC (gRNA2) for Pax2 mutation and GTGTCAGCAAAATCCTGGGC (gRNA1) and GCCAAACCTGCGGCGCAGGC (gRNA2) for Pax8 mutation.

## Electronic supplementary material

Supplementary information
