# Supplementary material for: An interspecies barrier to tetraploid complementation and chimera formation
Source: Sci Rep. 2018 Oct 16;8:15289. doi: 10.1038/s41598-018-33690-7 (PMC6191448; doi:10.1038/s41598-018-33690-7)
Supplement: Supplementary file 1 — Supplementary information [file 41598_2018_33690_MOESM1_ESM.pdf]

An interspecies barrier to tetraploid complementation and chimera formation

Tomoyuki Yamaguchi, Hideyuki Sato, Toshihiro Kobayashi , Megumi Kato-ito, Teppei Goto, Hiromasa Hara, Naoaki Mizuno, Ayaka Yanagida, Ayumi Umino, Sanae Hamanaka, Fabian Suchy, Hideki Masaki, Yasunori Ota, Masumi Hirabayashi, Hiromitsu Nakauchi

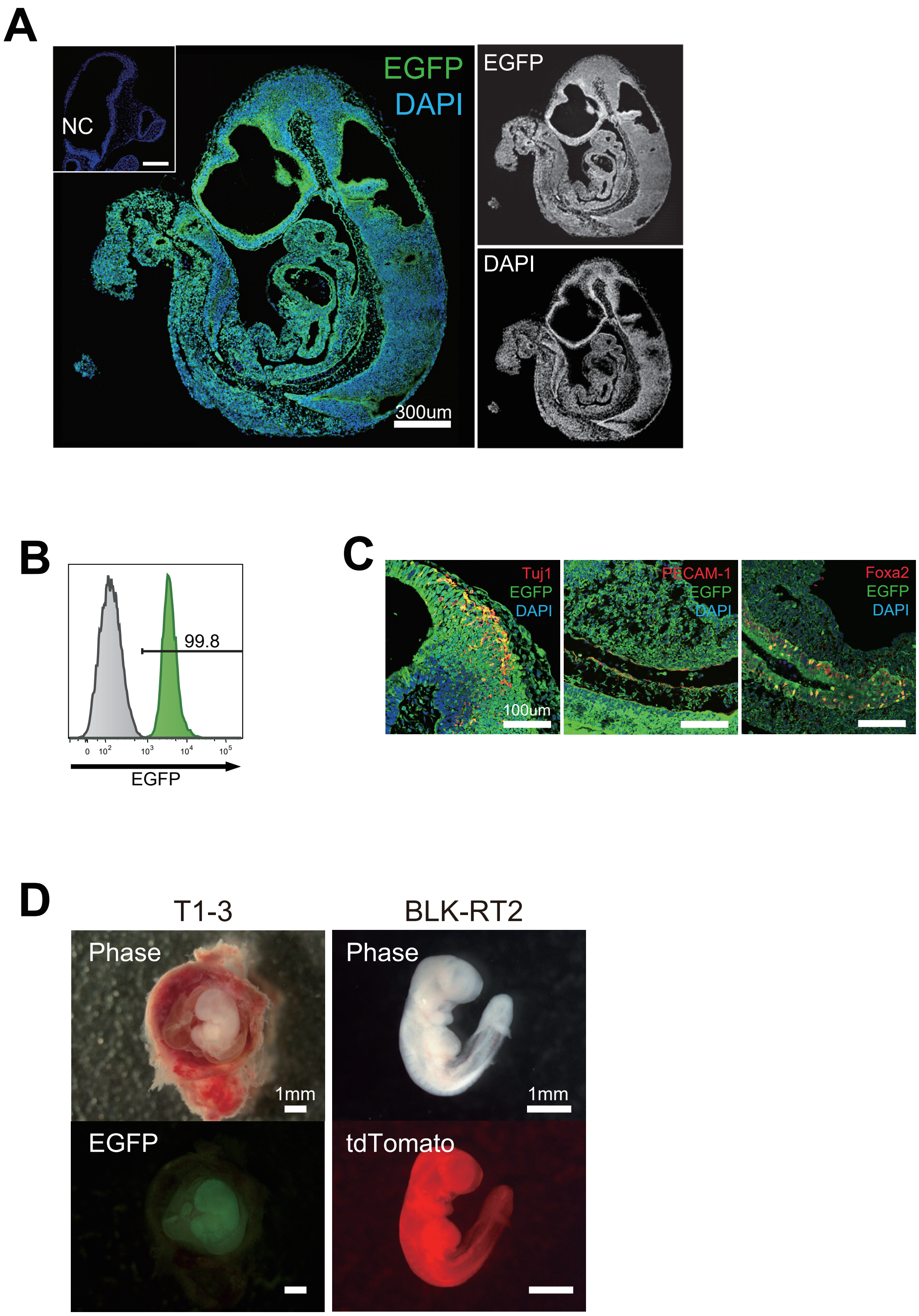
A
NC
EGFP
DAPI
300um
EGFP
DAPI
B
99.8
0
10^2
10^3
10^4
10^5
EGFP
C
Tuj1
EGFP
DAPI
100um
PECAM-1
EGFP
DAPI
Foxa2
EGFP
DAPI
D
T1-3
BLK-RT2
Phase
1mm
Phase
1mm
EGFP
tdTomato

A

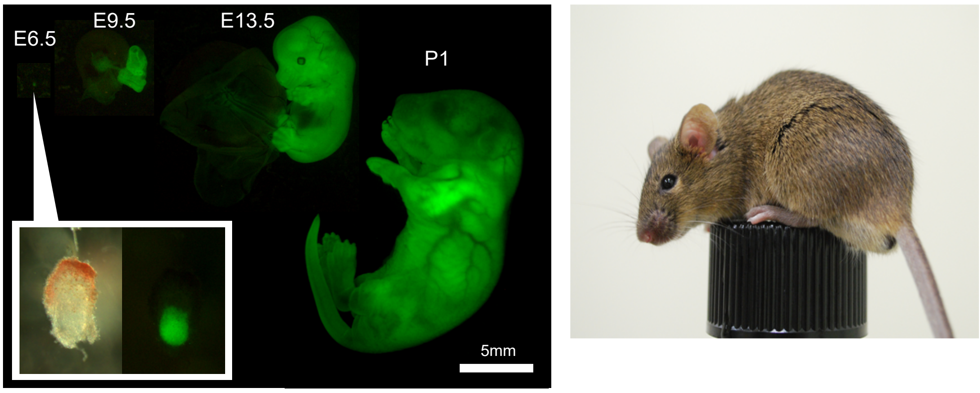

B

Donor cell name	Donor strain	Donor cell type	Stage of analysis	No. of implantation	No. of fetus (%)	No. of PSC-derived live fetuses or pups (%)
GT3.2	C57BL/6	iPSC	E6.5	20	13 (65)	11 (55)
			E9.5	22	12 (55)	12 (55)
			E13.5	16	5 (31)	5 (31)
			P1	24	2 (8)	2 (8)
K3	129sv x C57BL/6	ESC	P1 <	69	21 (30)	21 (30)

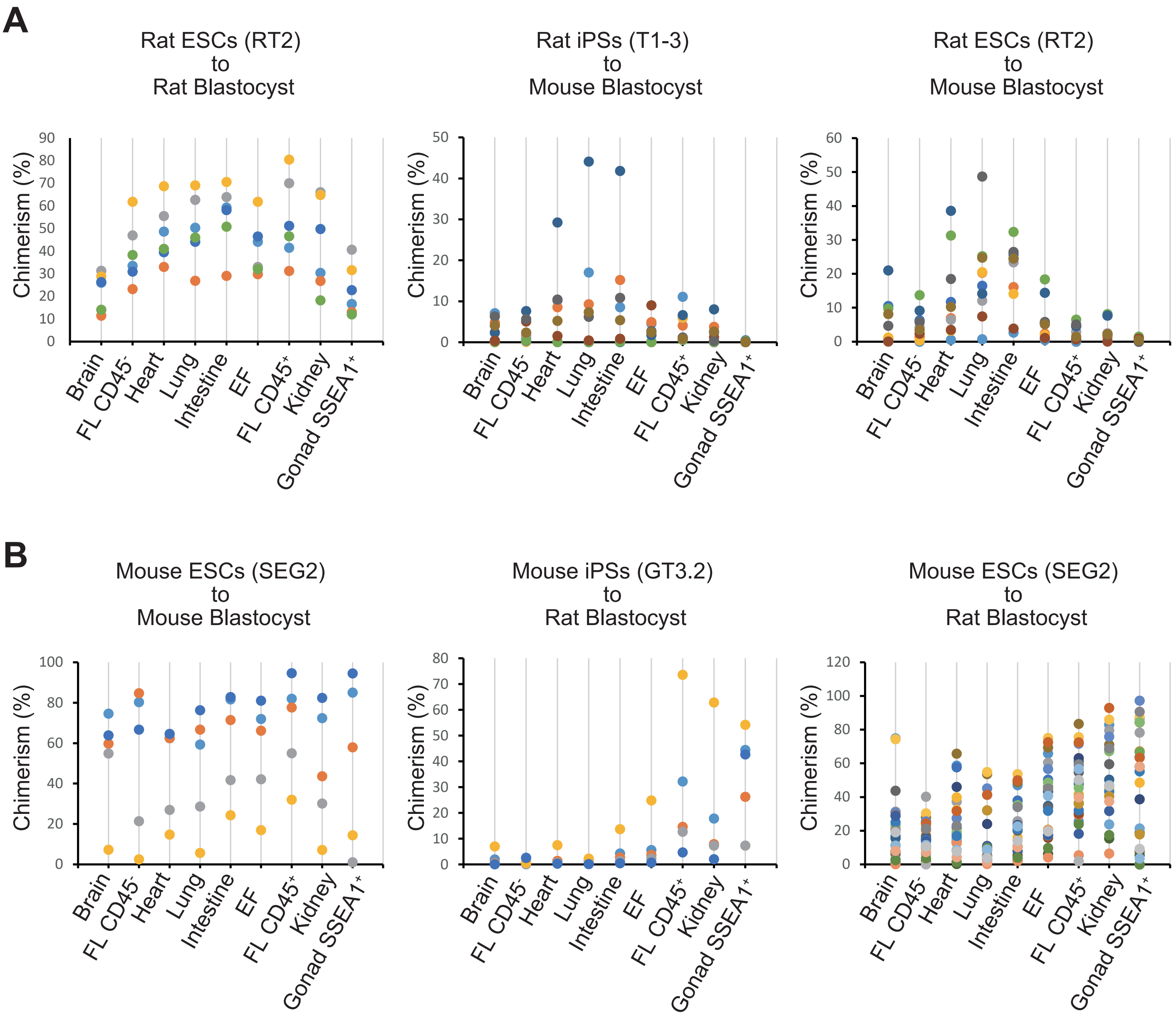
A
Rat ESCs (RT2)
to
Rat Blastocyst
Chimerism (%)
Brain
FL CD45-
Heart
Lung
Intestine
EF
FL CD45+
Kidney
Gonad SSEA1+
Rat iPSs (T1-3)
to
Mouse Blastocyst
Rat ESCs (RT2)
to
Mouse Blastocyst
B
Mouse ESCs (SEG2)
to
Mouse Blastocyst
Mouse iPSs (GT3.2)
to
Rat Blastocyst
Mouse ESCs (SEG2)
to
Rat Blastocyst

A

Chimerism (%) in various organ

	Brain	FL CD45$^-$	Heart	Lung	Intestine	EF	FL CD45$^+$	Kidney	Gonad SSEA1$^+$
Chimera No.1	60.7	6.37	49	47	28.6	30	2.29	N.D.	0.345
Chimera No.2	21	7.94	35.7	19.5	19.5	7.32	3.8	2.21	0.298
Chimera No.3	77.1	19.8	69.5	97.4	50.2	39.1	4.49	N.D.	1.44

B

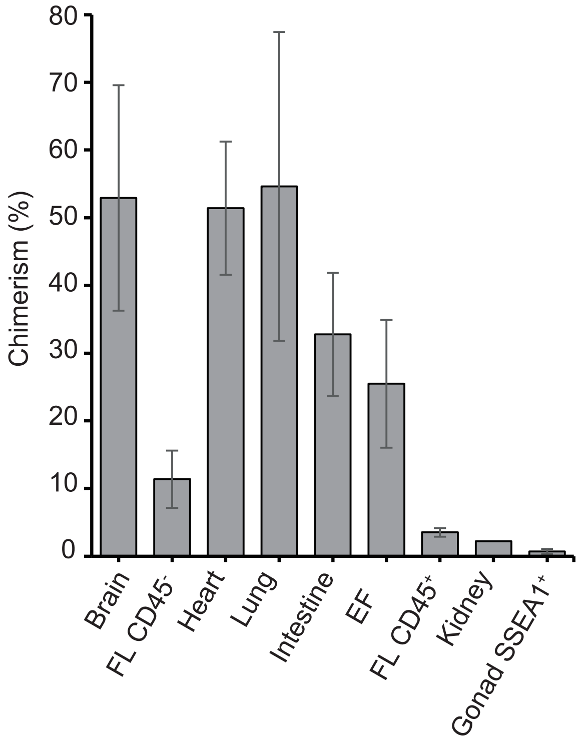

Supplementary Figure legends

Supplementary Figure 1. Development of rat PSC derived embryos in mouse tetraploid embryos

(A) Section of E9.5 rat iPSC derived embryo immunostained for EGFP with DAPI nuclear counterstaining. Scale bars: 300um. (B) FACS analysis using enzymatically digested E9.5 rat iPSC derived embryo. (C) Sections of rat iPSC derived embryo at E9.5 immunostained for EGFP with Tuj1, Pecam1, Foxa2, and DAPI nuclear counterstaining. Scale bars: 100um. (D) Bright field images (upper) and fluorescence images (lower) of degenerating E11.5 embryo generated by injection of rat iPSCs (T1-3) or ESCs (BLK-RT2). Scale bars: 1mm.

Supplementary Figure 2. Development of completely mouse iPSC derived mouse embryos generated via intraspecies tetraploid complementation

(A) Fluorescence images of E6.5, E9.5, E13.5, and post-natal day1 (P1) mouse iPSC derived embryos (left). Mouse iPSCs derived adult mouse (right). Scale bar: 5mm. (B) Results of intraspecies tetraploid complementation with mouse PSCs.

Supplementary Figure 3. Mouse or rat PSC derivative chimerism in the organs of

intra- and inter-species chimeras

(A) Individual chimerism of Figure 3A. Dots of same color represent same individuals.

(B) Individual chimerism of Figure 3B. Dots of same color represent same individuals.

Supplementary Figure 4. Rat ICM derivative chimerism in the organs of interspecies chimeras generated by injection of rat ICM into mouse tetraploid embryos

(A) ICM derivative chimerism in the organs of individual interspecies chimeras.

(B) Average ICM chimerism by organ, interspecies chimeras.
